# A chromosome-scale assembly for ‘d’Anjou’ pear

**DOI:** 10.1093/g3journal/jkae003

**Published:** 2024-01-08

**Authors:** Alan Yocca, Mary Akinyuwa, Nick Bailey, Brannan Cliver, Harrison Estes, Abigail Guillemette, Omar Hasannin, Jennifer Hutchison, Wren Jenkins, Ishveen Kaur, Risheek Rahul Khanna, Madelene Loftin, Lauren Lopes, Erika Moore-Pollard, Oluwakemisola Olofintila, Gideon Oluwaseye Oyebode, Jinesh Patel, Parbati Thapa, Martin Waldinger, Jie Zhang, Qiong Zhang, Leslie Goertzen, Sarah B Carey, Heidi Hargarten, James Mattheis, Huiting Zhang, Teresa Jones, LoriBeth Boston, Jane Grimwood, Stephen Ficklin, Loren Honaas, Alex Harkess

**Affiliations:** HudsonAlpha Institute for Biotechnology, Huntsville, AL 35806, USA; Department of Entomology and Plant Pathology, Auburn University, Auburn, AL 36849, USA; Department of Biological Sciences, Auburn University, Auburn, AL 36849, USA; Department of Biological Sciences, Auburn University, Auburn, AL 36849, USA; Department of Entomology and Plant Pathology, Auburn University, Auburn, AL 36849, USA; Department of Biological Sciences, Auburn University, Auburn, AL 36849, USA; Department of Biological Sciences, Auburn University, Auburn, AL 36849, USA; Department of Biological Sciences, Auburn University, Auburn, AL 36849, USA; Department of Biological Sciences, Auburn University, Auburn, AL 36849, USA; Department of Biological Sciences, Auburn University, Auburn, AL 36849, USA; Department of Biological Sciences, Auburn University, Auburn, AL 36849, USA; Department of Biological Sciences, Auburn University, Auburn, AL 36849, USA; Department of Biological Sciences, Auburn University, Auburn, AL 36849, USA; Department of Biological Sciences, University of Memphis, Memphis, TN 38152-3530, USA; Department of Biological Sciences, Auburn University, Auburn, AL 36849, USA; Department of Biological Sciences, Auburn University, Auburn, AL 36849, USA; Department of Biological Sciences, Auburn University, Auburn, AL 36849, USA; Department of Entomology and Plant Pathology, Auburn University, Auburn, AL 36849, USA; Department of Biological Sciences, Auburn University, Auburn, AL 36849, USA; Department of Biological Sciences, Auburn University, Auburn, AL 36849, USA; Department of Biological Sciences, Auburn University, Auburn, AL 36849, USA; Department of Biological Sciences, Auburn University, Auburn, AL 36849, USA; HudsonAlpha Institute for Biotechnology, Huntsville, AL 35806, USA; Physiology and Pathology of Tree Fruits Research Laboratory, USDA ARS, Wenatchee, WA 98801, USA; Physiology and Pathology of Tree Fruits Research Laboratory, USDA ARS, Wenatchee, WA 98801, USA; Physiology and Pathology of Tree Fruits Research Laboratory, USDA ARS, Wenatchee, WA 98801, USA; Department of Horticulture, Washington State University, Pullman, WA 99164-6414, USA; HudsonAlpha Institute for Biotechnology, Huntsville, AL 35806, USA; HudsonAlpha Genome Sequencing Center, HudsonAlpha Institute for Biotechnology, Huntsville, AL 35806, USA; HudsonAlpha Institute for Biotechnology, Huntsville, AL 35806, USA; HudsonAlpha Genome Sequencing Center, HudsonAlpha Institute for Biotechnology, Huntsville, AL 35806, USA; HudsonAlpha Institute for Biotechnology, Huntsville, AL 35806, USA; HudsonAlpha Genome Sequencing Center, HudsonAlpha Institute for Biotechnology, Huntsville, AL 35806, USA; Department of Horticulture, Washington State University, Pullman, WA 99164-6414, USA; Physiology and Pathology of Tree Fruits Research Laboratory, USDA ARS, Wenatchee, WA 98801, USA; HudsonAlpha Institute for Biotechnology, Huntsville, AL 35806, USA

**Keywords:** genome assembly, comparative genomics, PacBio HiFi, haplotype phased, whole-genome duplication

## Abstract

Cultivated pear consists of several *Pyrus* species with *Pyrus communis* (European pear) representing a large fraction of worldwide production. As a relatively recently domesticated crop and perennial tree, pear can benefit from genome-assisted breeding. Additionally, comparative genomics within Rosaceae promises greater understanding of evolution within this economically important family. Here, we generate a fully phased chromosome-scale genome assembly of *P. communis* ‘d’Anjou.’ Using PacBio HiFi and Dovetail Omni-C reads, the genome is resolved into the expected 17 chromosomes, with each haplotype totaling nearly 540 Megabases and a contig N50 of nearly 14 Mb. Both haplotypes are highly syntenic to each other and to the *Malus domestica* ‘Honeycrisp’ apple genome. Nearly 45,000 genes were annotated in each haplotype, over 90% of which have direct RNA-seq expression evidence. We detect signatures of the known whole-genome duplication shared between apple and pear, and we estimate 57% of d’Anjou genes are retained in duplicate derived from this event. This genome highlights the value of generating phased diploid assemblies for recovering the full allelic complement in highly heterozygous crop species.

## Teaser

Yocca *et al*. present a fully-phased chromosome-scale genome assembly of the European pear *P. communis* “d'Anjou”. This assembly will allow for genome-assisted breeding and will thus increase the pace of improvement for this relatively recently-cultivated, economically-important tree species. Additionally, comparative genomic studies within the Rose family (Rosaceae) may provide insight into the evolutionary history of the European pear. The genome assembly, annotation, and analyses were produced in a semester-long undergraduate and graduate course as part of the ACTG: American Campus Tree Genomes Initiative.

## Introduction

*Pyrus* L. is a genus in the family Rosaceae (subfamily Maloideae) comprising cultivated and wild pears. *Pyrus* is divided into 2 broad categories, the European and Asian pears, with their divergence estimated around 3–6 MYA (Wu *et al*. 2018). At least 26 species of *Pyrus* and 10 naturally occurring interspecific crosses are now found in Western and Eastern Asia, Europe, North Africa, and the Middle East (Bell and Itai, 2011). In 2021, the pear's value of utilized production in the United States reached $353 million (United States Department of Agriculture National Agricultural Statistics Service 2023). This makes pear one of the most cultivated pome fruits worldwide. One of the most important North American varieties of pear, the Anjou, also known as the Beurre d'Anjou or simply Anjou (*Pyrus communis* ‘d'Anjou’), is thought to have originated in Belgium, named for the Anjou region of France.

Over the last decade, several pear genomes have been sequenced and assembled using a variety of technologies. The first *Pyrus* genome sequenced in 2012 was the most commercially important Asian pear *Pyrus bretschneideri* Rehd. ‘Dangshansuli,’ using a combination of BAC-by-BAC sequencing and mate pair Illumina sequencing (Wu *et al*. 2013). Following that, European pear (*P. communis* ‘Bartlett’) was sequenced using Roche 454 (Chagné *et al*. 2014). In 2019, the *P. communis* genome was updated by sequencing the doubled-haploid ‘Bartlett’ cultivar using PacBio long reads and high-throughput chromosome conformation capture (Hi-C) technology (Linsmith *et al*. 2019). This assembly helped uncover duplicated gene models in previous assemblies that overassembled heterozygous regions. However, being a doubled-haploid, it still lacked an entire parental complement. A draft assembly and annotation for *P. communis* ‘d’Anjou’ was generated recently (Zhang *et al*. 2022), which was carefully annotated and revealed systematic differences in gene annotations across Rosaceae genomes. However, this assembly was also not phased, lacking information on allelic variants. Genomes are currently available for 5 of 26 *Pyrus* species in the Genome Database for Rosaceae (GDR; https://www.rosaceae.org/organism/26137) and for only a few of the thousands of recognized cultivars (Li *et al*. 2022).

Here, we sequenced and assembled a chromosome-scale reference genome for *P. communis* ‘d’Anjou’ using PacBio HiFi and Dovetail Omni-C sequencing. This genome was assembled as part of a semester-long undergraduate and graduate genomics course under the American Campus Tree Genomes (ACTG) initiative, where undergraduate and graduate students assemble, annotate, and publish culturally and economically valuable tree species. Here, we present a haplotype-resolved, chromosome-scale assembly and annotation of Anjou pear, place it in a phylogenetic context with other Rosaceae species, and show evidence of an ancient whole-genome duplication (WGD) event shared by cultivated apple and pear.

## Methods

### Genome sequencing

Tissue was acquired from Van Well Nursery as described in Zhang *et al.* (Zhang *et al*. 2022). The source material was labeled as the cultivar ‘d’Anjou.’ It should be noted we consider ‘Anjou’ and ‘Beurré d’Anjou’ as synonymous cultivar names. DNA was isolated from young leaf tissue using a standard CTAB approach (Doyle and Doyle 1987). Illumina TruSeq DNA PCR-free libraries were constructed from 1 μg of input DNA and sequenced on an Illumina NovaSeq6000 at HudsonAlpha Institute for Biotechnology. These short-reads were generated for plastid genome assembly as well as genome size estimation and postassembly assessment. Raw reads were assessed for quality using FASTQC v0.11.9 (Andrews *et al*. 2010). Then, low-quality reads were filtered out of the raw data by using *fastp* v0.12.4, allowing the generation of a statistical report with MultiQC 1.13.dev0 (Ewels *et al*. 2016). Nuclear genome size and ploidy were estimated using jellyfish v2.2.10 ((Marçais and Kingsford 2011; Ranallo-Benavidez *et al.* 2020)) to count *k*-mers and visualized in GenomeScope2.0 (Marçais and Kingsford 2011; Ranallo-Benavidez *et al.* 2020). For PacBio HiFi sequencing, ∼20 g of young leaf tissue from a ‘d’Anjou’ pear clone were collected and flash-frozen in liquid nitrogen. High molecular weight DNA was isolated from the young leaf tissue using a Circulomics Nanobind Plant Nuclei Big DNA kit (Baltimore, MD), with 4 g of input tissue and a 2 h lysis. DNA was tested for purity via spectrophotometry, quantified by Qubit dsDNA Broad Range, and size-selected on an Agilent Femto Pulse. DNA was sheared with a Diagenode Megaruptor and size-selected to roughly 25 kb on a BluePippin. A PacBio sequencing library was produced using the SMRTbell Express Template Prep Kit 2.0, and circular consensus sequencing (CCS) (HiFi) reads were produced on 2 8 M flow cells. PacBio HiFi read quality was assessed for read quality vs read distribution (Supplementary Fig. 1) using software *Pauvre* v0.2.3 (Schultz *et al.* 2019).

### Plastid genome assembly and annotation

The plastid genomes from 5 *Pyrus* individuals (Supplementary Table 3) were assembled using *NOVOPlasty* v4.3.1 (Dierckxsens *et al.* 2016), setting the expected plastid genome size to 130–170 kb and using the seed file provided (https://github.com/ndierckx/NOVOPlasty). The assembled plastid genomes were annotated using *GeSeq* v2.0.3 (Tillich *et al*. 2017) and visualized using *OGDRAW* v1.3.1 (Greiner *et al.* 2019).

### Genome assembly and scaffolding

Raw HiFi reads were assembled into contigs using *hifiasm* v0.16.0 (Cheng *et al*. 2021). To scaffold the ‘d’Anjou’ genome, 1 g of young leaf tissue was used as input for a Dovetail Omni-C library per manufacturer instructions (Dovetail Genomics, Inc.). The Omni-C library was sequenced on an Illumina NovaSeq6000 using paired-end 150 base-pair reads. To map the Omni-C data to our preliminary genome assembly, the Arima genomics pipeline was followed (https://github.com/ArimaGenomics/mapping_pipeline). Scaffolding was then performed using yet another Hi-C scaffolding tool (YaHS) with default parameters (Zhou *et al.* 2023). Omni-C contact maps were visualized using Juicebox version 1.11.08 (Durand *et al*. 2016). Several examples of likely misassembled regions were manually rearranged in Juicebox and documented in the Supplementary Methods. Genome completeness was assessed using compleasm v0.2.2 with the lineage “embryophyta_odb10” (Huang and Li 2023).

### Annotating repeats and transposable elements

Transposable elements (TEs) were predicted and annotated from the pear genome assembly using the Extensive de novo TE Annotator (EDTA) pipeline (v1.9.3) (Xu and Wang 2007; Ellinghaus *et al.* 2008; Xiong *et al*. 2014; Ou and Jiang 2019, 2018; Ou *et al*. 2019; Su *et al.* 2019; Shi and Liang 2019). EDTA parameters were set to the following: “--species others --step all --sensitive 1 --anno 1 --evaluate 1 --threads 4.” The coverage of genes and repeats in 1 Mb windows with a 100 Kb step was calculated using BEDTools version 2.30.0 (Quinlan and Hall 2010) and plotted onto the chromosomes using karyoploteR version 1.18.0 (Gel and Serra 2017).

### Structural variant analysis

First, assemblies were aligned using MUMmer (Marçais *et al*. 2018). Next, structural variants were characterized between genome assemblies using Assemblytics (Nattestad and Schatz 2016). More details are provided in the Supplementary Methods.

### Gene annotation

Protein-coding genes were annotated using MAKER2 (Holt and Yandell 2011). Arabidopsis Araport 11 proteins and 7 *P. communis* ‘d’Anjou’ RNA-seq libraries were used as evidence (Cheng *et al*. 2017). RNA-seq libraries are available on the National Center for Biotechnology Information (NCBI) Sequence Read Archive (SRA) under accession PRJNA791346. One round of evidence-based annotation was performed and used to iteratively train ab initio prediction models through both SNAP and Augustus. More details are provided in the Supplementary Methods.

### RNA-seq analyses

RNA-seq reads were retrieved from the NCBI SRA under accession PRJNA791346. Reads were adapter trimmed using the BBMap “bbduk.sh” script (https://sourceforge.net/projects/bbmap/). Gene expression was quantified using Kallisto (Bray *et al*. 2016). Clustering was performed using the “heatmap()” function in R (R Core Team 2022). More details are provided in the Supplementary Methods.

### Comparative genomic analyses

Putative synteny constrained orthologs between *P. communis* ‘d’Anjou,’ *Malus domestica* ‘Honeycrisp,’ and *Prunus cerasus* ‘Montmorency’ were identified using the JCVI utilities library compara catalog ortholog function (Tang et al. 2008). Genome assemblies and annotations were retrieved from the Genome Database for Rosaceae. Synonymous substitution rates were calculated using a custom Ka/Ks pipeline (https://github.com/Aeyocca/ka_ks_pipe). Briefly, orthologs were aligned using MUSCLEv3.8.31 (Edgar 2004), and PAL2NAL v14 was used to convert the peptide alignment to a nucleotide alignment, and Ks values were computed between gene pairs using codeml from PAML v4.9 with parameters specified in the control file found in the GitHub repository listed above (Yang 1997; Suyama *et al.* 2006).

## Results

### Nuclear genome assembly

**Fig. 1.**
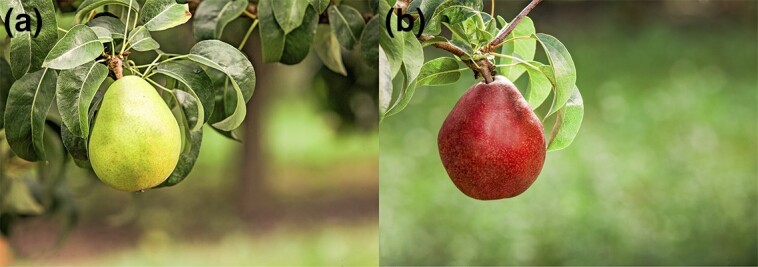
Pear fruit photographs. Photographs of green Anjou fruit (a) and Red Anjou fruit (b). Photos were provided by USA Pears.

We generated several types of sequencing data to assemble and annotate the Anjou genome (Fig. 1). Given an estimated genome size of ∼550 Mb (Niu *et al*. 2020), we generated 113× coverage of Illumina shotgun data, 66× of PacBio HiFi data, and 190× of Omni-C data per haplotype. Genomescope estimated a *k*-mer-based genome size of ∼495 Mb, 46.79% repeated sequences, and 1.79% heterozygosity (Supplementary Fig. 1). We assessed the quality of our HiFi reads using Pauvre indicating high-quality libraries and a read length distribution centered around 15 kb (Supplementary Fig. 2). Our mean and median read lengths were 15,555 and 14,758 bp, while the longest read was 49,417 bp long.

The final assembly is haplotype-resolved with 17 chromosomes per haplotype. Chromosomes were oriented according to the *M. domestica* ‘Honeycrisp’ assembly (Khan *et al*. 2022). The final assembly consisted of nearly 540 Mb per haplotype with >93% of the raw contig assemblies contained in the 17 chromosomes (Supplementary Fig. 3). The contig N50s for haplotypes 1 and 2 respectively were 14.7 and 13.4 Mb, while the scaffold N50s were 29.6 Mb. We found >99% complete BUSCOs in each haplotype with over 30% of them present in duplicate, reflecting the WGD experienced by the Maleae lineage ∼45 MYA (Xiang *et al*. 2017). Over 99% of our Illumina reads were properly mapped back to our assembly. *k*-mer-based completeness between Illumina reads and the final assembly demonstrated high-quality values (36.16) and low error rates (0.0002423) for both haplotypes.

### Chloroplast assembly

**Fig. 2.**
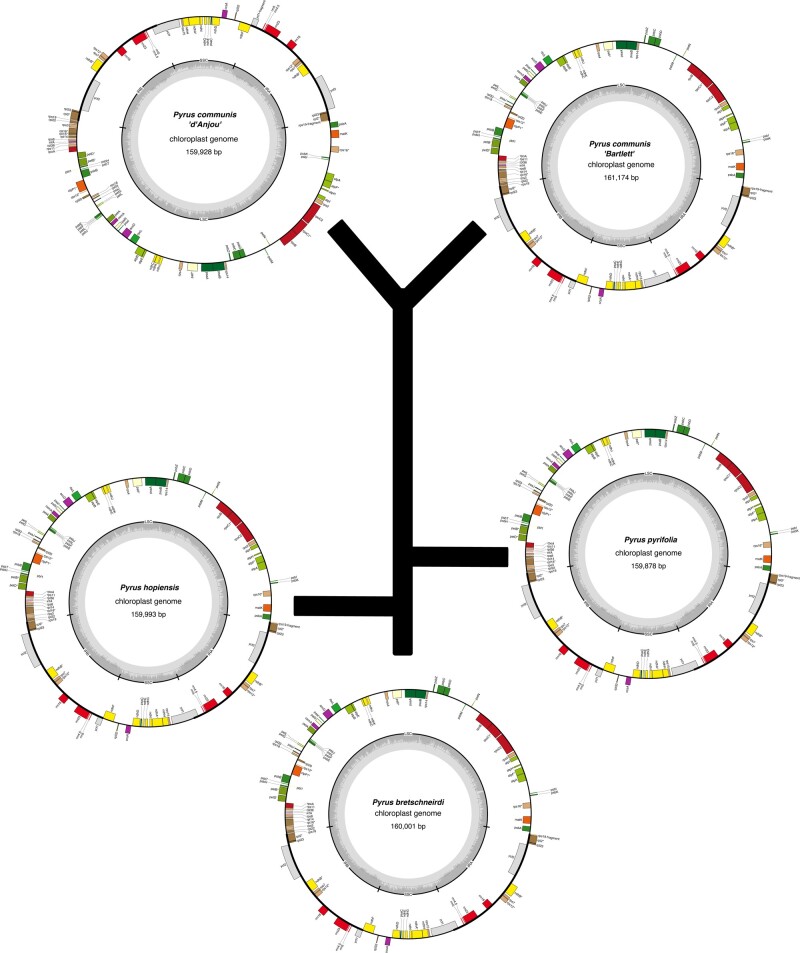
Chloroplast assemblies and phylogeny. Chloroplast genomes of assorted pear cultivars—assemblies and annotations. Plastid assemblies were carried out using *NOVOPlasty* v4.4.1 and annotated using *GeSeq* v2.0.3. Phylogenetic relationships were estimated using maximum likelihood under the generalized time reversible model.

We also assembled the chloroplast of *P. communis* ‘d’Anjou’ along with 4 other *Pyrus* species or accessions (Supplementary Table 3 and Fig. 4; Fig. 2). The chloroplast genomes were similar in size, ranging from 159 to 161 kb, and consisted of a large single-copy region, small single-copy region, and 2 inverted repeats for each species. *Pyrus* as a genus consists of 2 major genetic groups: European and Asian (Zheng *et al*. 2014). *Pyrus hopeiensis*, *Pyrus pyrifolia*, and *Pyrus bretscheirderi* are all considered Asian species. We estimated phylogenetic relationships between our chloroplast assemblies and found both representatives of *P. communis* sister to each other consistent with expectations.

**Fig. 3.**
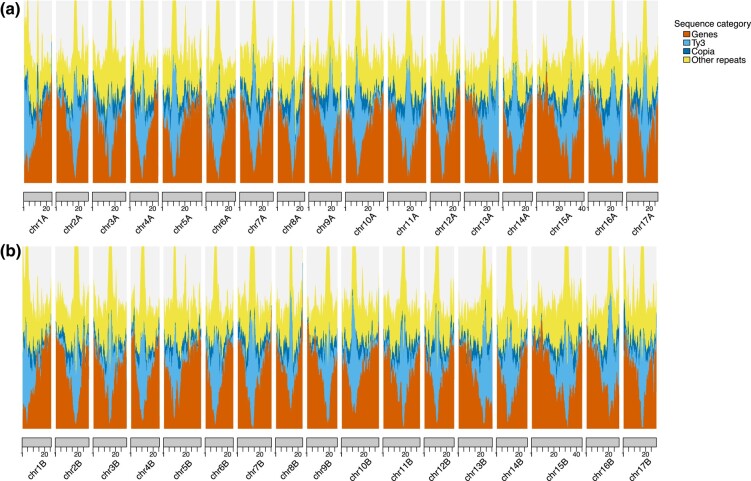
Distributions of genomic elements. Density of genomic elements across our assembly. Feature densities are calculated in 1 Mb windows with a 100 kb step size. Features on haplotype 1 are listed in a), and those on haplotype 2 are listed in b). Feature distributions are stacked in the order: Genes (bottom distribution), Ty3 TEs (second lowest distribution), Copia TEs (second highest distribution), and other repeat elements annotated by EDTA (highest distribution). Numbers along the x-axis correspond to position along the chromosome (Mb).

TEs are important components of plant genomes, contributing to genome size variation, gene family evolution, and transcriptional novelty (Lu *et al*. 2019; Quadrana 2020). Repetitive elements were annotated using EDTA (Ou *et al*. 2019) (Table 1). A total of 39–42% of each haplotype consisted of repetitive elements. The majority of these elements by length were long terminal repeat (LTR) retrotransposons accounting for ∼32% of each haplotype. These elements are most abundant around the putative centromeres but are also ubiquitous in gene-rich regions (Fig. 3). Terminal inverted repeats (TIRs) were also abundant and dominated by mutator elements (∼3.4% of each haplotype).

**Table 1.** Summary of repeat elements annotated by EDTA.

Repeat type	Hap	Count	bp masked	% Masked	Repeat type	Hap	Count	bp masked	% Masked
LTR Ty1	1	31,417	29,651,485	5.6	LTR Ty1	2	30,811	29,080,309	5.73
LTR Ty3	1	52,870	65,248,004	12.32	LTR Ty3	2	51,619	65,330,713	12.88
LTR unknown	1	52,617	44,783,539	8.46	LTR unknown	2	60,287	50,732,038	10
TIR CACTA	1	20,714	7,389,362	1.4	TIR CACTA	2	19,593	7,081,084	1.4
TIR mutator	1	75,530	18,368,328	3.47	TIR mutator	2	71,859	17,304,544	3.41
TIR PIF harbinger	1	26,889	9,561,615	1.81	TIR PIF harbinger	2	25,649	9,164,523	1.81
TIR Tc1 mariner	1	1,950	713,551	0.13	TIR Tc1 mariner	2	1,857	567,099	0.11
TIR hAT	1	14,789	4,479,323	0.85	TIR hAT	2	13,724	4,267,786	0.84
LINE	1	1,494	720,397	0.14	LINE	2	1,409	710,461	0.14
Non-LTR unknown	1	242	304,682	0.06	Non-LTR unknown	2	215	279,820	0.06
Helitron	1	25,911	8,267,980	1.56	Helitron	2	29,480	9,716,313	1.92
Other repeat regions	1	83,566	21,068,202	3.98	Other repeat regions	2	87,157	21,406,735	4.22
Total	1	387,989	210,556,468	39.78	Total	2	393,660	215,641,425	42.52

LTR, long terminal repeat; TIR, terminal inverted repeat; PIF, P instability factor; LINE, long interspersed nuclear element; Hap, haplotype; Bp, base pairs.

Each haplotype was independently annotated with expression evidence, Arabidopsis protein evidence, and ab initio gene prediction using the MAKER pipeline (Supplementary Methods and Table 4). We annotated a total of 44,839 genes in haplotype A and 44,561 genes in haplotype B, which is similar to the number of genes annotated in *M. domestica* ‘Honeycrisp’ (50,105). Gene density was highest on chromosome arms and was inversely related to the density of TEs (Fig. 3).

There were several structural variants between our 2 haplotypes (Table 2). We characterized 13,421 variants within 50–10,000 base pairs between the haplotypes, totaling almost 32 Mb of sequence. Repeat expansion and contractions were the largest classes of structural variant. Insertions and deletions also affected nearly 6 Mb of sequence between haplotypes. Between *P. communis* ‘d’Anjou’ and *P. communis* ‘Bartlett,’ 14,946 variants affected 26 Mb of sequence. The total amount of sequence affected is lower than that observed between ‘d’Anjou’ haplotypes. This may simply be due to a more complete assembly for both Anjou haplotypes relative to the ‘Bartlett’ assembly.

**Table 2.** Structural variants between 50 and 10,000 bp identified by Assemblytics.

Reference	Query	Variant type	# Variants	# Bases affected
‘d’Anjou’ Hap1	‘d’Anjou’ Hap2	Indel	4,297	6,000,228
‘d’Anjou’ Hap1	‘d’Anjou’ Hap2	Repeat	8,711	24,943,411
‘d’Anjou’ Hap1	‘Bartlett’	Indel	5,739	4,439,368
‘d’Anjou’ Hap1	‘Bartlett’	Repeat	8,910	11,571,098

Indel is short for “insertion/deletion.”

### Comparative genomics and polyploidy

Rosaceae as a plant family contains several important crops such as pear, apple, peach, cherry, and blackberry. Comparative genomics between these crops may allow functional genomics in 1 species to be translated to others. Therefore, we compared the genomes of 3 of these important crops: *P. communis* ‘d’Anjou’ (pear), *M. domestica* ‘Honeycrisp’ (apple (Khan *et al*. 2022)), and *P. cerasus* ‘Montmorency’ (cherry (Goeckeritz *et al*. 2023)). Both our assembled haplotypes were highly collinear with each other and with apple. We identified 40,567 orthologs between pear haplotypes, 30,340 orthologs between pear haplotype 1 and apple, and 20,526 orthologs to *P. cerasus* ‘Montmorency’ consistent with pear's divergence with apple postdating that to cherry.

Apple and pear share a WGD occurring after their divergence with cherry (Xiang *et al*. 2017). Our results show they both demonstrate a high percentage (>⅓) of duplicated BUSCO genes as well as 17 chromosomes, almost double the Amygdaloideae base chromosome count of 9 (Hodel *et al*. 2021). Therefore, we infer apple and pear retain much of their genome in duplicate. Across all genes within *P. communis* ‘d’Anjou,’ ∼57% are classified as having a syntenic paralog retained from this WGD event (Supplementary Table 5).

**Fig. 4.**
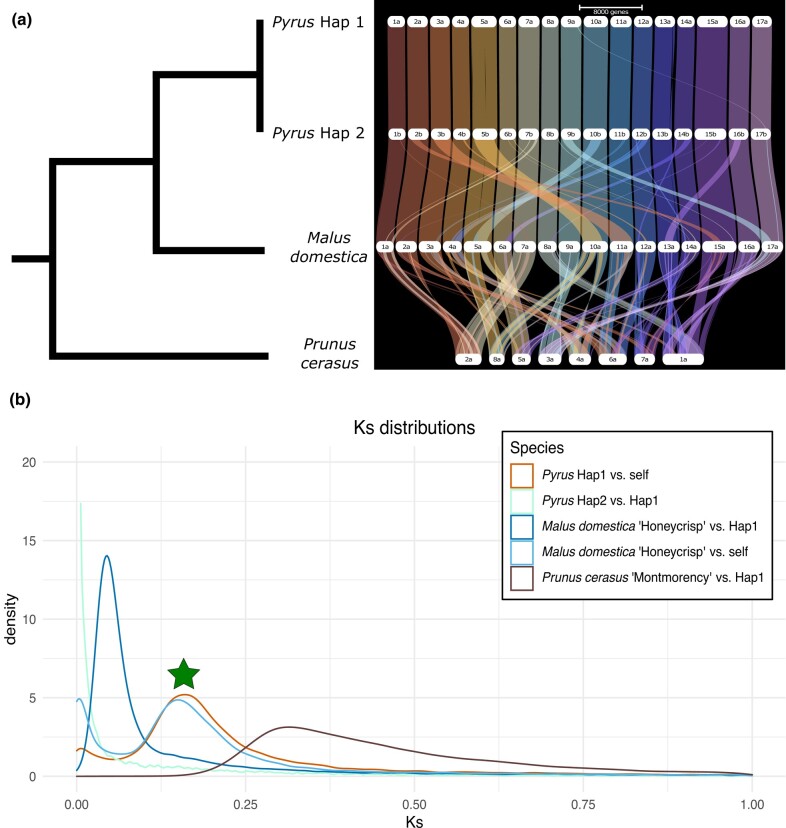
Ribbon plot and **K**s distributions. a) A phylogenetic tree with known relationships between 4 assemblies. To the right is a ribbon plot based on gene synteny created with GENESPACE (Lovell *et al*. 2022). b) A density plot showing the distribution of synonymous substitution rates (Ks) between genome-wide gene pairs. The shared WGD event is denoted by a green star. All comparisons are to *P. communis* ‘d’Anjou’ haplotype 1 except for the “*M. domestica* self” comparison. Abbreviations are as follows: *Pyrus* Hap1, *P. communis* ‘d’Anjou’ haplotype 1; *Pyrus* Hap2, *P. communis* ‘d’Anjou’ haplotype 2.

‘Montmorency’ is a tetraploid formed from a hybridization between different *Prunus* species after their divergence with the common ancestor of apple and pear. Therefore, we only compared the “A” subgenome to our assemblies. As expected, each cherry “A” subgenome scaffold was syntenic with ∼2 pear and apple scaffolds (Fig. 4a). Additionally, there were blocks in pear syntenic with 2 regions of apple that are likely regions retained from the last WGD event. There were likely further karyotype changes since the divergence of Malineae and cherry as the syntenic blocks are not entirely retained nor perfectly paired in 1:2 ratios. However, there remains high collinearity with these genomes suggesting future translation of functional genomics across species.

The distribution of synonymous substitution rates (Ks) across gene pairs indicates the divergence between them as gene pairs will accumulate synonymous substitutions over time (Yang and Nielsen 2000; Senchina *et al*. 2003). We see orthologs between haplotypes 1 and 2 in our assembly have a Ks distribution centered near 0 as expected for allelic copies of genes that are still segregating within the species. Comparing haplotype 1 to itself identifies gene pairs that are retained from the most recent WGD event. We see this distribution is higher than that of gene pairs between *Pyrus* and *Malus* suggesting this WGD event occurred before the divergence of these species. Additionally, comparing *M. domestica* with itself shows a distribution similar to that of the “*Pyrus* self” comparison, as expected reflecting a shared WGD event or, at the very least, a different WGD event occurring around the same time (Fig. 4b; star). This distribution is lower than that compared with *P. cerasus* as this WGD event postdates the divergence of the cherry and apple/pear lineages.

### Gene expression

**Fig. 5.**
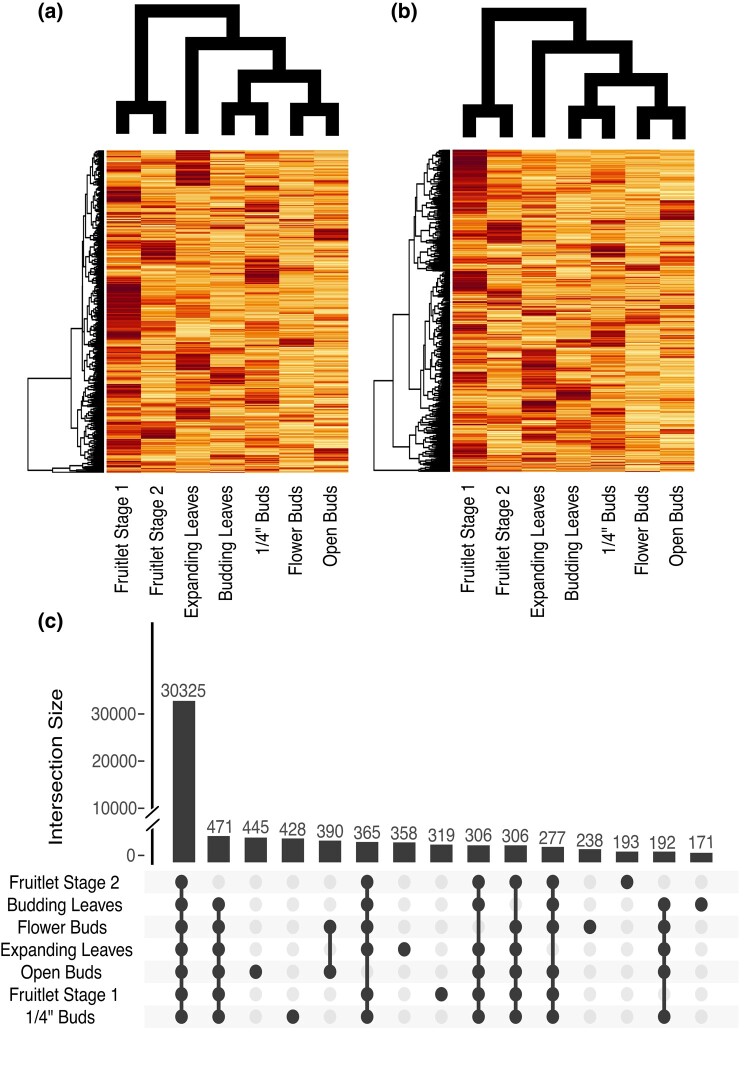
Gene expression characterization. Heatmaps and UpSet plot of gene expression. Cladograms represent the relationships between libraries through hierarchical clustering. A total of 1000 genes are displayed that show expression in each tissue and have the highest expression variance. a) Haplotype 1. b) Haplotype 2. c) UpSet plot of expression across tissues for haplotype 1. Genes were considered expressed if they had a TPM value above 1. Note the break in the y-axis.

We quantified gene expression across 7 tissues (Table 3). We found expression evidence for ∼33–35,000 gene models per tissue. Most gene models were expressed in fruitlet stage 1, and the least were expressed in fruitlet stage 2 suggesting dynamic gene expression across fruit development. There was evidence of gene expression in at least a single tissue for 40,734 gene models, while 2,152 genes were expressed in only a single tissue (average of 307 genes per tissue). Our expression data were generated to assist genome annotation and are only single replicates. We therefore cannot perform differential expression analyses. We instead performed hierarchical clustering of gene expression (Fig. 5). We see stable clustering across haplotypes and find similar tissues cluster together. For example, our 2 fruit libraries clustered with each other. We generated an UpSet plot showing the 15 largest intersects of genes expressed >1 transcript per million (TPM; Fig. 5). The largest intersect was genes expressed >1 TPM in every tissue queried. The top 15 intersects, however, included each of the 7 tissue-specific categories. Open buds had the most tissue-specific genes (445), while budding leaf–specific genes had the least (171).

**Table 3.** Expression characteristics of *P. communis* ‘d’Anjou.’

Tissue	Hap	Genes expressed	Median TPM	Tissue	Hap	Genes expressed	Median TPM
Budding leaves	1	33,594	84.97	Budding leaves	2	33,470	88.00
Expanding leaves	1	34,469	119.7	Expanding leaves	2	34,380	122.0
Flower buds	1	34,138	71.34	Flower buds	2	34,082	73.3
Fruitlet stage 1	1	34,923	193	Fruitlet stage 1	2	34,797	200
Fruitlet stage 2	1	33,227	96.4	Fruitlet stage 2	2	33,107	100.0
Open buds	1	34,463	72.0	Open buds	2	34,372	74.02
¼” buds	1	34,718	108.3	¼” buds	2	34,513	111.00

Hap, haplotype; TPM, transcripts per million reads.

## Conclusion

We assembled a chromosome-scale phased genome assembly for cultivated European pear as part of the ACTG: American Campus Tree Genomes initiative where students assemble, annotate, and publish iconic tree genomes in semester courses. PacBio HiFi reads coupled with Dovetail Omni-C resulted in a high-quality assembly, displaying high *k*-mer completeness, quality scores, synteny with available assemblies, and recovery of universal single-copy orthologs. This assembly revealed thousands of structural variants between haplotypes which are of great importance to future pear breeding efforts as structural variants disrupt recombination. Comparative analyses between other members of the Rosaceae family demonstrated deeply conserved synteny and recovered evidence for a 45 million-year-old WGD event. Gene expression across several tissue types was largely conserved, but thousands of genes also constrained themselves to a single tissue. Further characterization of pear germplasm will accelerate breeding gains not only within pear but potentially across multiple Rosaceous crops. Lastly, we highlight the utility of generating such genomes as part of semester courses and the training opportunities that it provides.

## Supplementary Material

jkae003_Supplementary_Data

## Data Availability

Data used to generate this assembly are deposited in the NCBI SRA under BioProject PRJNA992953. Gene expression data are available separately under BioProject PRJNA791346. Custom scripts used throughout are available on GitHub (https://github.com/Aeyocca/dAnjou_genome_MS). Genome assembly and annotation files are available on GDR (https://www.rosaceae.org/Analysis/17650423) and on the NCBI SRA under accession numbers PRJNA1047602 and PRJNA1047603. Supplemental material available at G3 online.
