# Supplementary material for: A chromosome-scale assembly for ‘d’Anjou’ pear
Source: G3 (Bethesda). 2024 Jan 8;14(3):jkae003. doi: 10.1093/g3journal/jkae003 (PMC10917493; doi:10.1093/g3journal/jkae003)
Supplement: jkae003_Supplementary_Data [file jkae003_supplementary_data.zip › Supplemental_Figures_G3-2023-404689.pdf]

Supplementary Figures

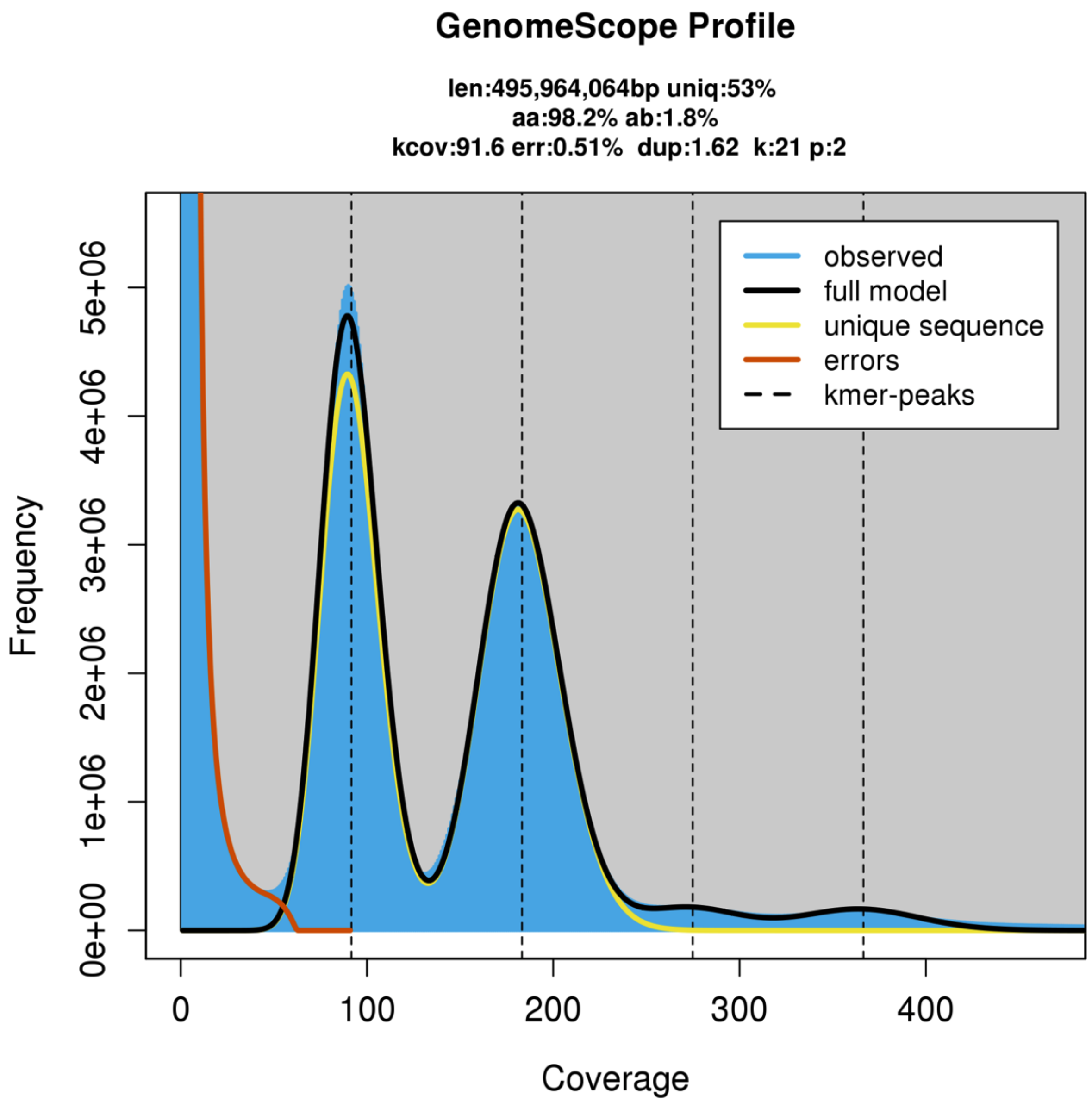

Figure S1: GenomeScope output for ‘d’Anjou’ short-read data. GenomeScope *k*-mer (k=21) profile plot showing there are two major diploid peaks. The tall peak at 91.6X coverage indicates high heterozygosity in this genome.

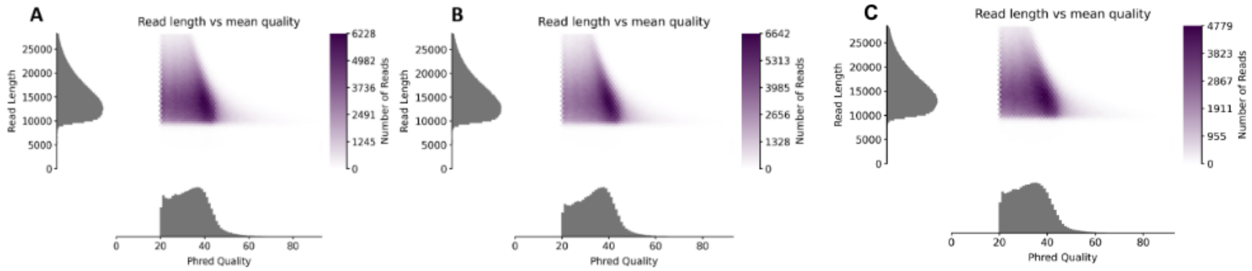

Figure S2: Phred quality analysis of three Pacbio HiFi cells; (A) m64017_211217_051206, (B) m64017_211218_161111, and (C) m64017_211210_084732

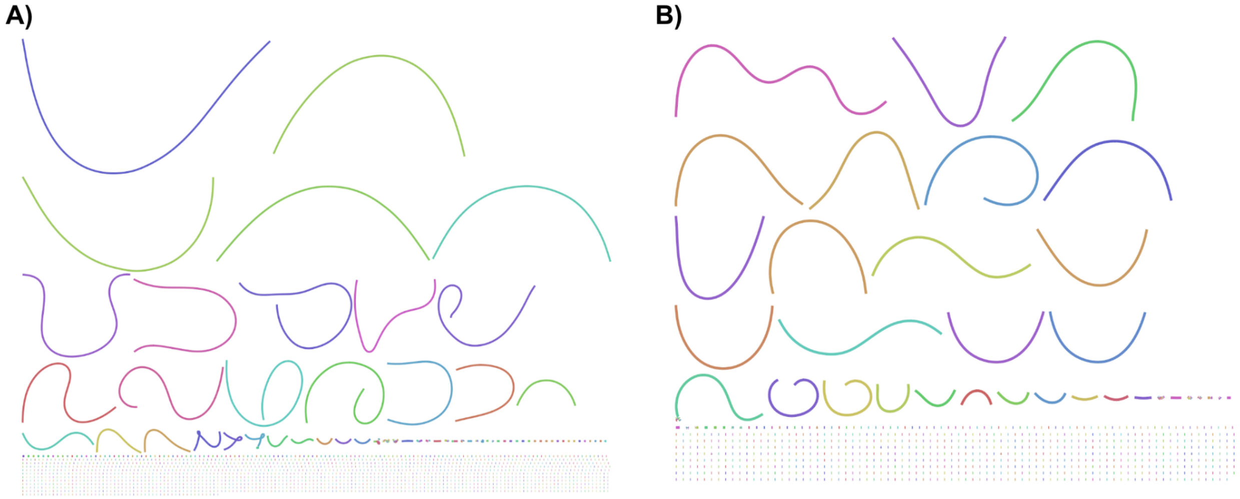

Figure S3: Bandage plots (Wick et al. 2015) for haplotype 1 (A) and haplotype 2 (B).

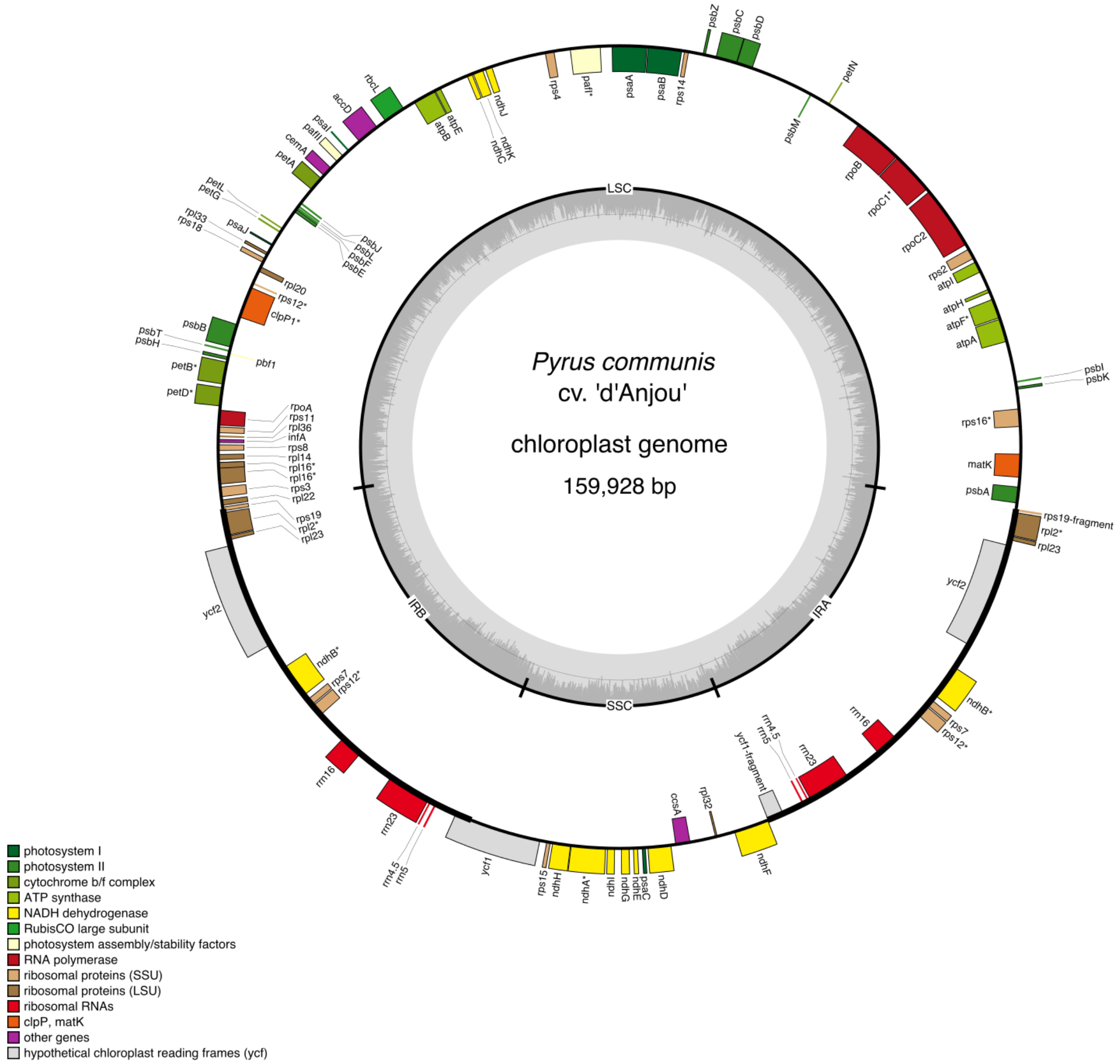

Figure S4: The 'd'Anjou' pear chloroplast genome assembly and annotation. Plastid assembly was carried out using *NOVOPlasty* v4.4.1 and annotated using *Ge-Seq* v2.0.3.

Platform	Number of reads	Number of bases (Gb)	Median length (bp)
Illumina NovaSeq 6000	831,658,352	124.8	150
Dovetail Omni-C	256,666,668	38.5	150
PacBio HiFi	4,660,447	72.5	14,758

Table S1: Summary of sequencing reads used for genome assembly.

**Genome size property ***	**min**	**max**
Homozygous (%)	98.19%	98.21%
Heterozygous (%)	1.79%	1.81%
Genome Haploid Length (bp)	493,204,035	493,809,912
Genome Repeat Length (bp)	230,747,204	231,030,666
Genome Unique Length (bp)	262,456,831	262,779,247
Model Fit (%)	61.87	95.25
Read Error Rate (%)	0.51	0.51
Repeats (%)	46.79	46.79

*measured from GenomeScope2.0 with k=21.

Table S2: Characteristics of 'd'Anjou' pear based on the short-read *k*-mer profile

**Species**	**Source**	**Number of bases (Gb)**	**SRAID**	**# of assembled contigs**	**# of base-pairs**
Pyrus hopeiensis HB-1	(Y. Li et al. 2021)	16.34	SRR14318823	3	159993
Pyrus pyrifolia	(M.-Y. Zhang et al. 2021)	50.04	DRR385062	4	159875
Pyrus communis "Bartlett DH"	(Linsmith et al. 2019)	29.27	SRR10030340	5	161148
Pyrus x *bretschneideri*	(Y. Li et al. 2021)	21.65	SRR14318827	3	160001
Pyrus communis "d'Anjou"	This publication		NA	1	159928

Table S3: Source of data for chloroplast assemblies.

Haplotype	# Genes	# Transcripts	Annotated bases	BUSCO string
1	44558	44839	68,452,305	C:97.1%[S:63.1%,D:34.0%],F:1.3%,M:1.6%,n:1614
2	44349	44561	68,084,531	C:97.6%[S:64.3%,D:33.3%],F:0.9%,M:1.5%,n:1614

Table S4: Annotation statistics. Abbreviations are as follows: C (complete), S (single-copy), D (duplicated), F (fragmented), M (missing), n (number).

Duplication type	Haplotype	# of genes	% of genes
Singleton	1	5726	12.77
Dispersed	1	6810	15.19
Proximal	1	2865	6.39
Tandem	1	3844	8.57
WGD or segmental	1	25594	57.08
Singleton	2	5818	13.06
Dispersed	2	6780	15.21
Proximal	2	2839	6.37
Tandem	2	4041	9.07
WGD or segmental	2	25083	56.29

Table S5: Duplication classification. WGD stands for “Whole Genome Duplication”
