# Supplementary material for: A chromosome-scale assembly for ‘d’Anjou’ pear
Source: G3 (Bethesda). 2024 Jan 8;14(3):jkae003. doi: 10.1093/g3journal/jkae003 (PMC10917493; doi:10.1093/g3journal/jkae003)
Supplement: jkae003_Supplementary_Data [file jkae003_supplementary_data.zip › Supplemental_Material_G3-2023-404689.pdf]

Supplementary Methods

Assembly, annotation, and analysis of the *Pyrus communis* cv. “d’Anjou” version 2 reference genome. Most filenames are listed as “v1” internally throughout this document, although another assembly existed (Zhang et al. 2022). Our assembly was thus renamed to “v2” in April 2023.

1. Assembly

1.1 Initial contig assembly

An initial assembly was generated using hifiasm and 35x depth of Omni-C per haplotype. Data were generated according to the Methods listed in the main manuscript.

```
#hifiasm version 0.16.1-r375
hifiasm -o Pyrus_PB_35x_Omni_v1.asm -t40 \
--h1 Pyrus_OmniC_35x_R1.fastq \
--h2 Pyrus_OmniC_35x_R2.fastq \
Pyrus_HiFi_all.fq
```

This primary assembly step using 40 threads took ~17 hours and peaked around 175Gb of RAM.

Next, the GFA file output from hifiasm was converted to a FASTA formatted file.

```
awk '/^S/{print ">"$2;print $3}'
Pyrus_PB_35x_Omni_v1.asm.hic.hap1.p_ctg.gfa >
Pyrus_PB_35x_Omni_v1.asm.hic.hap1.p_ctg.fasta
```

1.2 Short contig removal and contig polishing

Contigs less than 50kb long were dropped, and then the assembly was polished with racon. Racon corrects potential base-pair assembly errors by mapping long-reads back to an assembly. The raw HiFi reads were mapped to the initial assembly using minimap2.

```
python drop_short_seqs.py --input
Pyrus_PB_35x_Omni_v1.asm.hic.hap2.p_ctg.fasta --trashbin
Pyrus_PB_35x_Omni_v1.asm.hic.hap2.p_ctg.trashbin_50kb.fasta --output
Pyrus_PB_35x_Omni_v1.asm.hic.hap2.p_ctg.50kb.fasta --length 50000

```

```
#minimap2 version 2.17-r941

minimap2 -a -t 30 \
Pyrus_PB_35x_Omni_v1.asm.hic.hap1.p_ctg.fasta \
Pyrus_HiFi_all.fq \
> Pyrus_35x_HiFi_mapped_hap1.sam

#racon version 1.5.0

~/racon/build/bin/racon -t 30 \
Pyrus_HiFi_all.fq Pyrus_35x_HiFi_mapped_hap1.sam \
Pyrus_PB_35x_Omni_v1.asm.hic.hap1.p_ctg.50kb.fasta \
> Pyrus_PB_35x_Omni_v1.asm.hic.hap1.p_ctg.50kb.racon.fasta
```

1.3 Omni-C mapping and scaffolding

Omni-C reads were mapped to the polished assembly to prepare for Yet another Hi-C Scaffolder (YaHS) according to the Arima Genomics mapping pipeline. Accessory scripts for the Arima Genomics pipeline can be found here https://github.com/ArimaGenomics/mapping_pipeline.

```
#bwa index on cmd line
#bwa index -a bwtsw -p
Pyrus_PB_35x_Omni_v1.asm.hic.hap1.p_ctg.50kb.racon.bwa_idx
Pyrus_PB_35x_Omni_v1.asm.hic.hap1.p_ctg.50kb.racon.fasta
#took 10 minutes

CPU=50
READ_FILE="/cluster/home/ayocca/01_Rosaceae_Evo/Pyrus/Pyrus_OmniC_35x
_R1.fastq"
BWA_IDX="Pyrus_PB_35x_Omni_v1.asm.hic.hap1.p_ctg.50kb.racon.bwa_idx"
RAW_OUT="Pyrus_hic_mapped_raw_R1.bam"
FILT_OUT="Pyrus_hic_mapped_filt_R1.bam"

bwa mem -t $CPU $BWA_IDX $IN_DIR/$READ_FILE \
| samtools view -@ $CPU -Sb \
- > $RAW_OUT

#echo "### Step 2.A: Filter 5' end (1st)"
samtools view -h $RAW_OUT | \
perl /cluster/home/ayocca/mapping_pipeline-master/filter_five_end.pl
| \
samtools view \
```

```
-Sb - > $FILT_OUT
```

The two read pairs were merged, a MapQ filter of 10 was applied, and duplicates were marked with picard tools in accordance with the Arima Genomics pipeline.

```
perl
/cluster/home/ayocca/mapping_pipeline-master/two_read_bam_combiner.pl
\
${BAM_ONE} ${BAM_TWO} samtools \
$MAPQ_FILTER | samtools view -bS -t $ref_faidx - | \
samtools sort -@ $CPU -T ~/tmp -o ${OUT}_merge

echo "adding useless readgroup"
java -Xmx4G -Djava.io.tmpdir=temp/ -jar $PICARD
AddOrReplaceReadGroups \
INPUT=${OUT}_merge OUTPUT=${OUT}_RG ID="SRA_tmp" LB="SRA_tmp"
SM="SRA_tmp" \
PL=ILLUMINA PU=none

java -Xmx30G -XX:-UseGCOverheadLimit -Djava.io.tmpdir=temp/ -jar
$PICARD \
MarkDuplicates INPUT=${OUT}_RG \
OUTPUT=${OUT} \
METRICS_FILE=${OUT}_metrics_file.txt \
TMP_DIR=$TMP_DIR ASSUME_SORTED=TRUE \
VALIDATION_STRINGENCY=LENIENT REMOVE_DUPLICATES=TRUE

samtools index ${OUT}
perl /cluster/home/ayocca/mapping_pipeline-master/get_stats.pl \
${OUT} > ${OUT}.stats
echo "Finished Mapping Pipeline through Duplicate Removal"

#final output file from this code block =
Pyrus_hic_mapped_filt_sort_markdup_hap1.bam
```

YaHS was run, then the contact maps were visualized in Juicebox (Durand et al. 2016).

```
/cluster/home/ayocca/Alan_bin/yahs/yahs \
Pyrus_PB_35x_Omni_v1.asm.hic.hap1.p_ctg.50kb.racon.fasta \
Pyrus_hic_mapped_filt_sort_markdup_hap1.bam \
```

-o Pyrus_hap1
```

```
/path/to/yahs/juicer pre -a -o Pyrus_hap1 Pyrus_hap1.bin
Pyrus_hap1_scaffolds_final.agp
Pyrus_PB_35x_Omni_v1.asm.hic.hap1.p_ctg.50kb.racon.fasta.fai
>Pyrus_hap1.log 2>&1
```

```

java -jar -Xmx10G
/path/to/juicer/juicer_tools.1.9.9_jcuda.0.8.jar pre
Pyrus_hap1.txt Pyrus_hap1.hic <(echo “assembly 540812361“)
```

The `540812361` value comes from the Pyrus_hap1.log file.

1.4 Misjoin identification and correction

Various misjoins and potential errors in the assembly were manually inspected and edited through a variety of means, using the contact maps, dotplots, and visual inspection. Here we detail all of the major edits made to the assembly and provide examples.
```

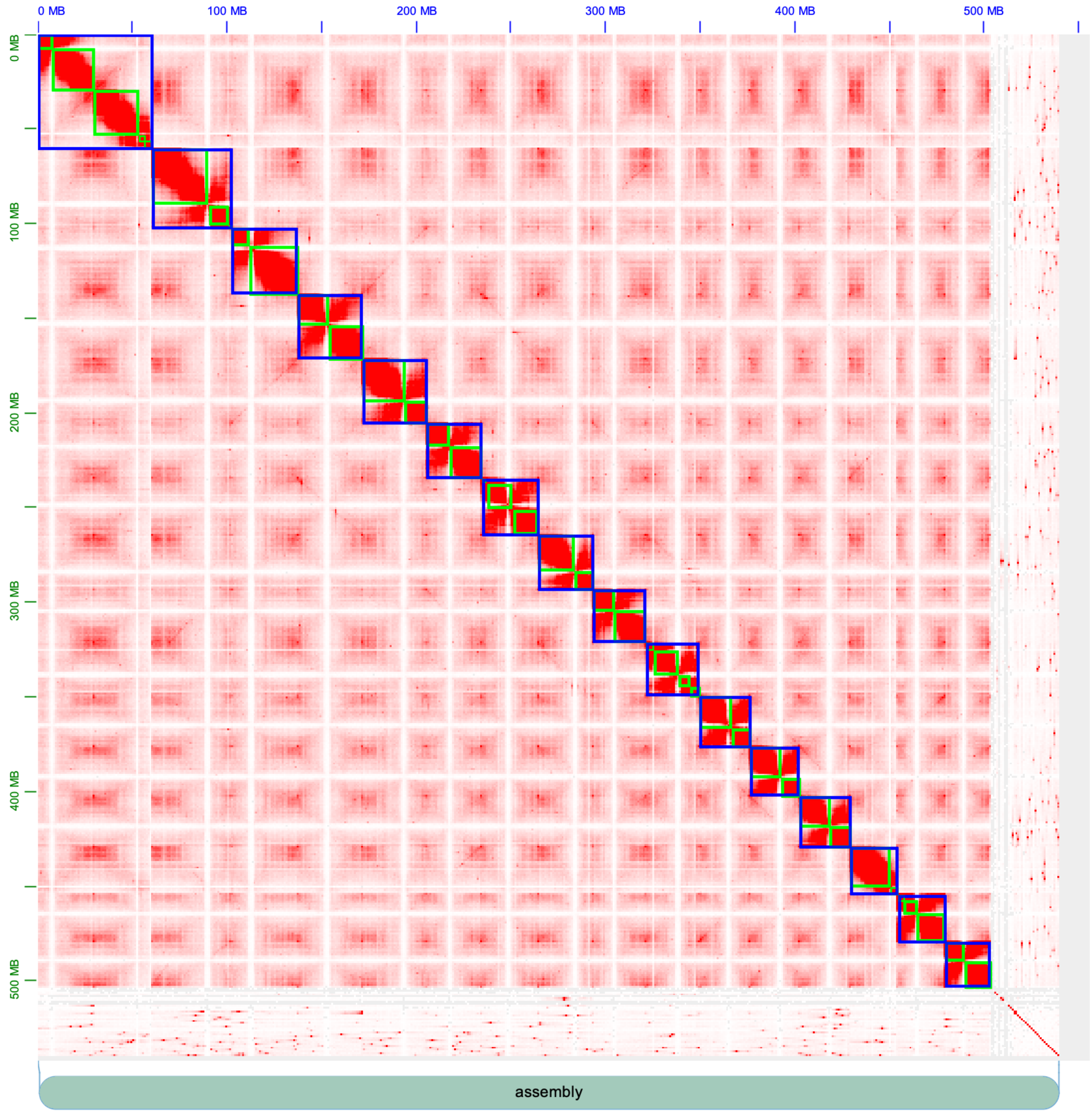

Figure SM1: Omni-C contact map of Pyrus_PB_35x_Omni_v1.asm.hic.hap1.p_ctg.50kb.racon.fasta

The initial Omni-C contact map for haplotype 1 is shown above. Scaffold 1 is a misjoin of two chromosomes as it is larger than any other scaffold and has little contact between the upper left and lower right contigs (delineated by green boxes). Scaffold 1 was therefore manually split and exported into the assembly file `Pyrus_hap1.review_11_09_22.assembly`.

Dot plots between haplotypes were generated using Gepard (https://github.com/univieCUBE/gepard). These supported evidence that `hap1_scaff1` was a misjoin between `hap2_scaff7` and `hap2_scaff10`, since the hypothesized misjoined

scaffold aligns to two separate scaffolds in haplotype 2 and is about twice the length of any other scaffold.

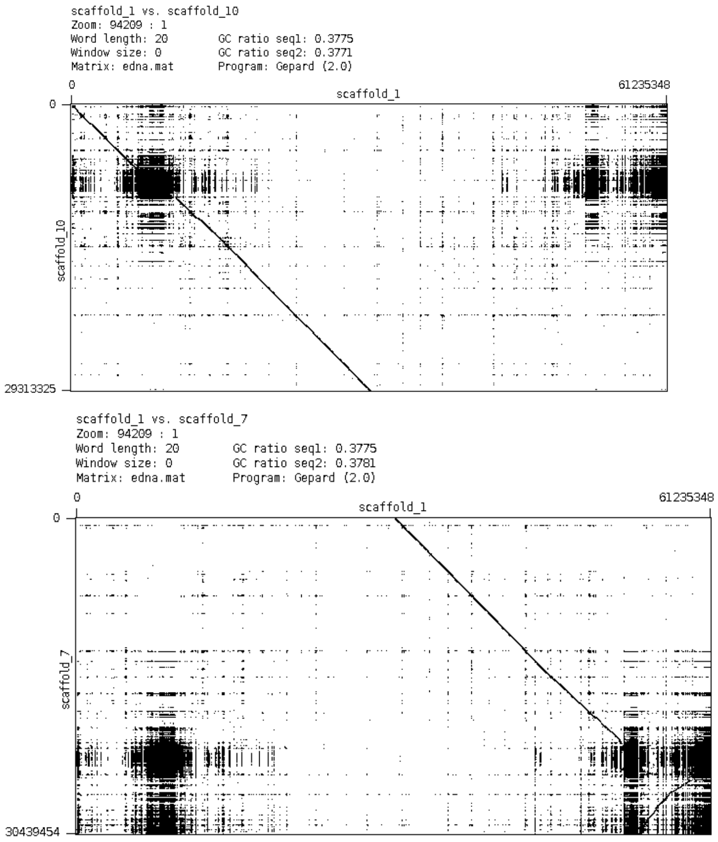

Figure SM2: Dot plots between Pyrus_PB_35x_Omni_v1.asm.hic.hap1.p_ctg.50kb.racon.fasta haplotype 1 and haplotype 2 of the same assembly. The x-axis represents haplotype 1 scaffold 1 in top and bottom plots. The y-axis of the top plot represents haplotype 2 scaffold 10. The y-axis of the bottom plot represents haplotype 2 scaffold 7.

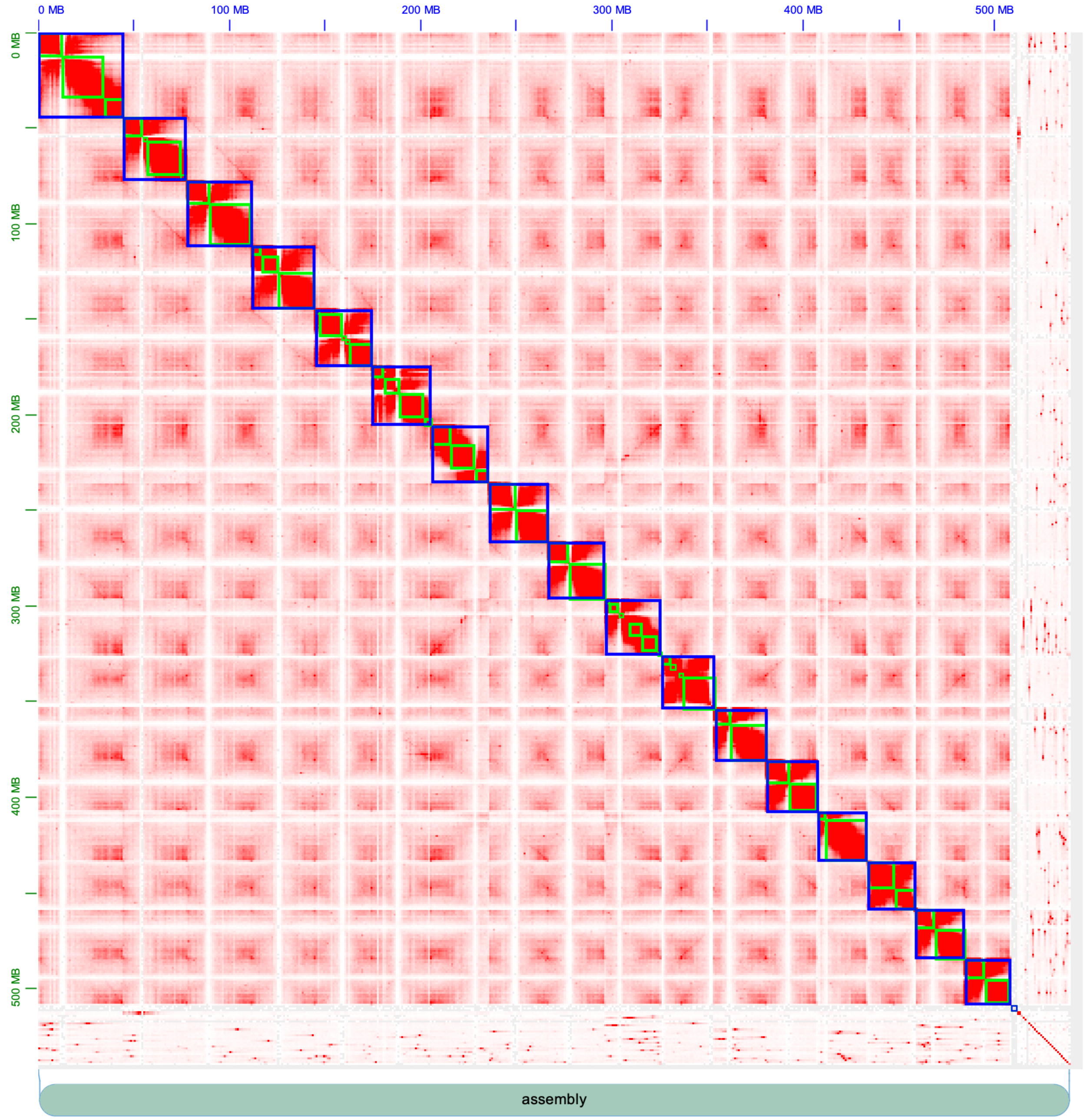

Figure SM3: Omni-C contact map of Pyrus_PB_35x_Omni_v1.asm.hic.hap2.p_ctg.50kb.racon.fasta after correcting the major chromosoma misjoin.

The assembly file produced by YaHS following manual curation was then converted into a FASTA formatted file.

```
#yahs version 1.2a.2
```

```
/path/to/yahs/juicer post -o Pyrus_hap1
Pyrus_hap1.review_11_09_22.assembly Pyrus_hap1.liftover.agp
../Pyrus_PB_35x_Omni_v1.asm.hic.hap1.p_ctg.50kb.racon.fasta
```

Gepard was used to generate dot plots to assign homologous chromosomes between haplotypes and to check for other assembly errors.

The scaffolds were also aligned to *Malus domestica* “Honeycrisp” to label chromosomes with their proper homologs.

```
java -cp
/path/to/miniconda3/envs/genome_tools/share/gepard/dist/Gepard-2.1.ja
r org.gepard.client.cmdline.CommandLine -seq ../hap1_scaff2.fasta
../hap2_scaff7.fasta -word 20 -outfile hap1_scaff2_hap2_scaff7_2.png
-matrix
~/miniconda3/envs/genome_tools/share/gepard/resources/matrices/edna.m
at
```

The dot plots between dAnjou haplotypes suggested entire chromosome arm inversions which are likely assembly errors as scaffolding software can struggle with highly repetitive regions such as centromeres. `hap1_scaff13` and `hap2_scaff11` are homologous chromosomes. Their dot plots show an inversion in chromosome arms relative to each other. Dot plots to *M. domestica* “Honeyrcisp” were generated to identify in which haplotype the likely assembly error occurred in.

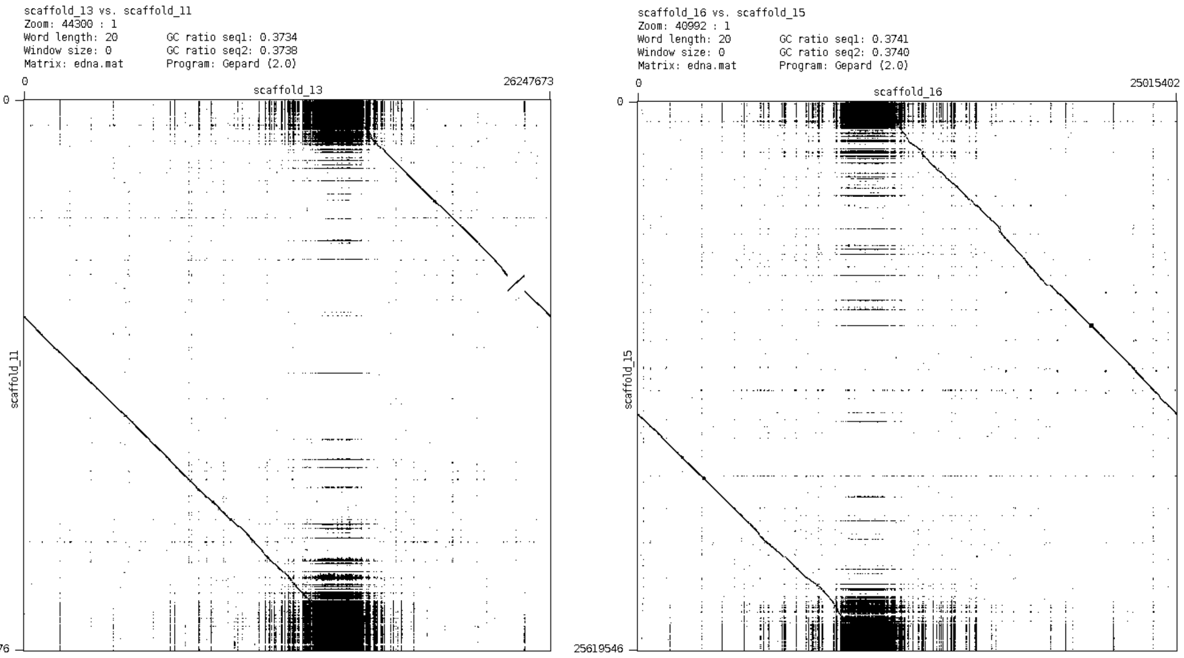

Figure SM4: Dot plots between Pyrus_PB_35x_Omni_v1.asm.hic.hap2.p_ctg.50kb.racon.fasta and Pyrus_PB_35x_Omni_v1.asm.hic.hap1.p_ctg.50kb.racon.fasta. The left plot shows haplotype 2 scaffold 11 (y-axis) against haplotype 1 scaffold 13 (x-axis). The right plot shows haplotype 2 scaffold 15 (y-axis) against haplotype 1 scaffold 16 (x-axis).

Dot plots to “Honeycrisp” confirmed this was a haplotype 2 specific misassembly.

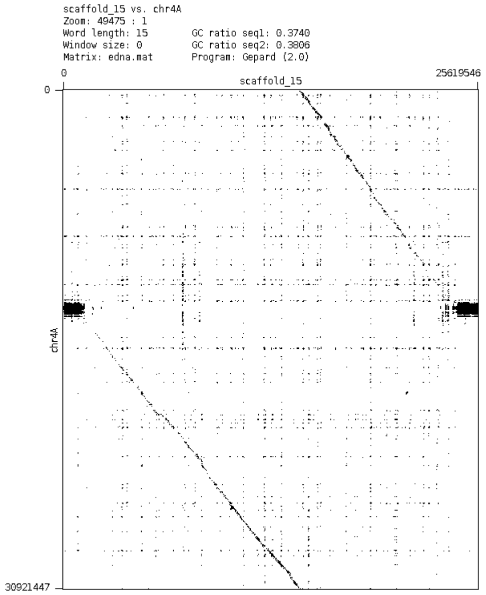

Figure SM4: Dot plots between Pyrus_PB_35x_Omni_v1.asm.hic.hap2.p_ctg.50kb.racon.fasta and Malus_x_domestica_Honeycrisp_HAP1_v1.1.a1.fasta. The x-axis represents *Pyrus* scaffold 15 and the y-axis represents *Malus* chromosome 4A.

Based on the dot plots, there were three hypothesized assembly errors.

Scaffold	Issue
Pyrus hap2_scaff11	Centromere in telomere
Pyrus hap2_scaff15	Centromere in telomere
Pyrus hap2_scaff7	Small-arm inversion

Table SM1: Hypothesized assembly errors based on dot plot visualizations.

These three assembly errors were manually corrected in Juicebox.

The fasta file was regenerated following manual correction.

```
/path/to/yahs/juicer post -o Pyrus_hap2
Pyrus_hap2.review_11_10_22.assembly Pyrus_hap2.liftover.agp
../Pyrus_PB_35x_Omni_v1.asm.hic.hap2.p_ctg.50kb.racon.fasta
```

Dotplots of the corrected scaffolds were generated and no longer suggested assembly errors.

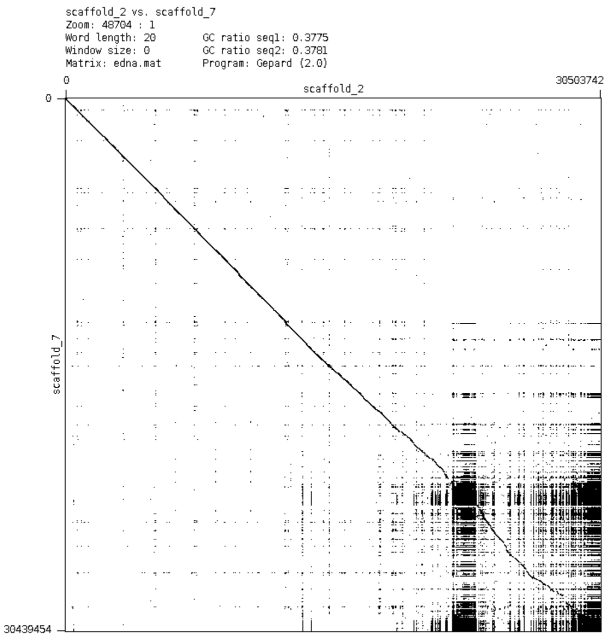

Figure SM5: Dotplot between a corrected haplotype 2 scaffold 7 (y-axis) against haplotype 1 scaffold 2 (x-axis).

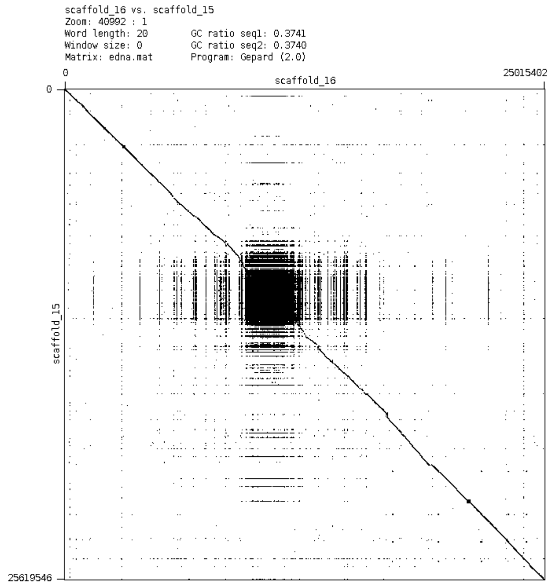

Figure SM6: Dotplot between a corrected haplotype 2 scaffold 15 (y-axis) against haplotype 1 scaffold 16 (x-axis).

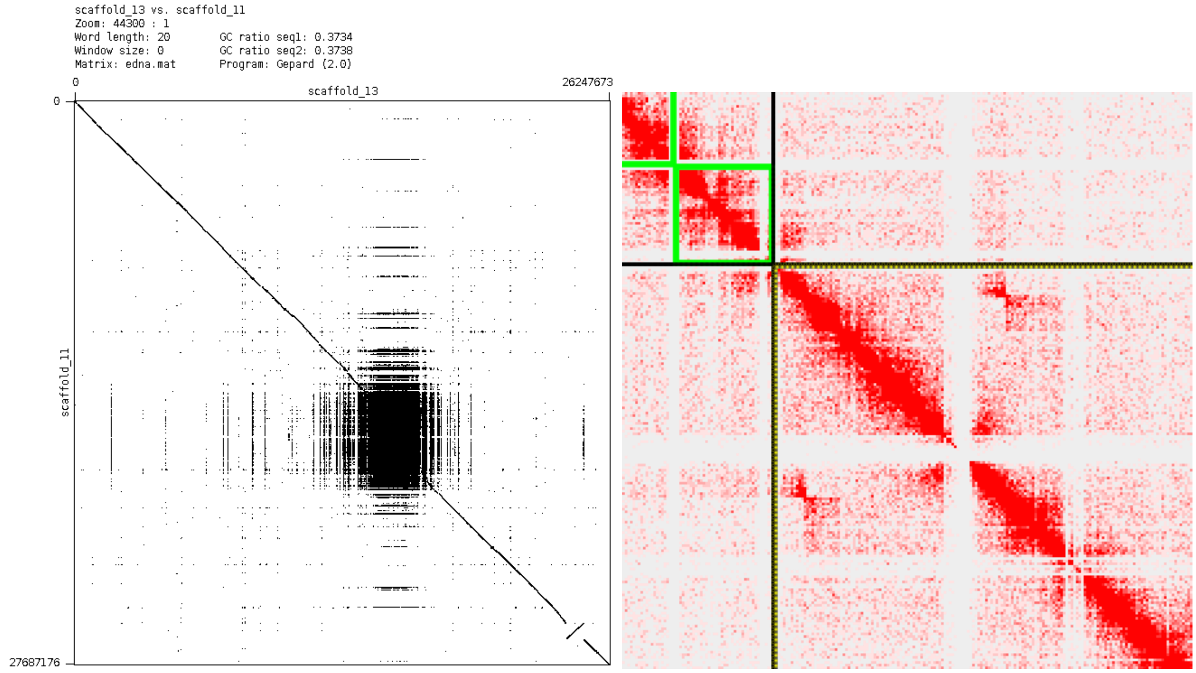

Figure SM7: (right) dotplot of haplotype 2 scaffold 11 (y-axis) against haplotype 1 scaffold 13 (x-axis). (right) Omni-C contact map of haplotype 2 zoomed in on the apparent inversion identified from the dotplot on the left. Images are not to scale.

The presence of a “bowtie” motif in the Omni-C contact map suggested this inversion is an assembly error in haplotype 2. This inversion was also manually corrected in Juicebox.

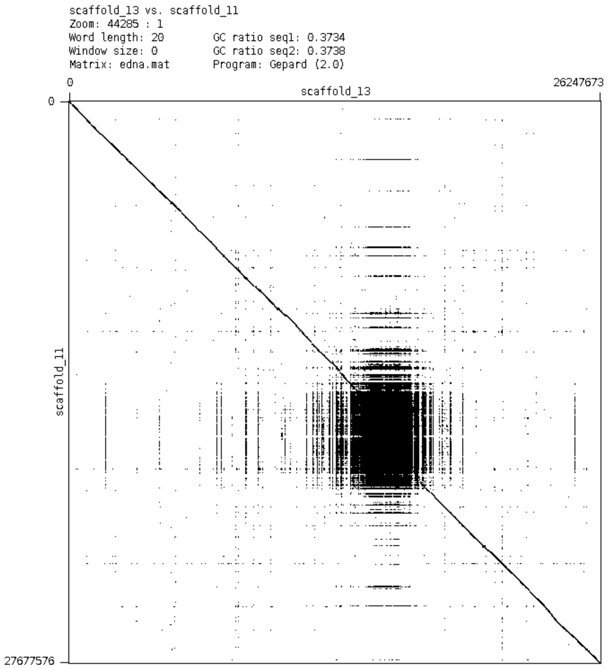

Figure SM8: dotplot of haplotype 2 scaffold 11 (y-axis) against haplotype 1 scaffold 13 after adjusting the apparent inversion on haplotype 2 in juicebox

Scaffolds were reverse complemented in Juicebox to orient them with *M. domestica* "Honeycrisp".

The list of scaffolds for haplotype 1 which needed reverse complemented were as follows: 1, 5, 6, 8, 10, 12, 13, 14, 15, 17.

The list of scaffolds for haplotype 2 which needed reverse complemented were as follows: 1, 4, 5, 6, 9, 10, 11, 12, 17

The scaffolds were re-numbered to match their homologous chromosomes in *M. domestica* "Honeycrisp".

```
~/Alan_bin/perl/rename_fa.pl -i hap2_HC_hap2_trans.txt -f
hic_maps/Pyrus_hap2_v1.FINAL.fa -o Pyrus_hap2_v1.rename.fasta
```

Omni-C data were remapped to the renumbered and reoriented version of the assembly.

```
/cluster/home/ayocca/Alan_bin/yahs/yahs \
../../Pyrus_hap1_v1.2.rename.fasta \
../../Pyrus_hic_mapped_filt_sort_markdup_hap1_v1.2.bam \
-o Pyrus_hap1_v1.2
```

```
/path/to/yahs/juicer pre -a -o Pyrus_hap1 Pyrus_hap1.bin
Pyrus_hap1_scaffolds_final.agp
Pyrus_PB_35x_Omni_v1.asm.hic.hap1.p_ctg.50kb.racon.fasta.fai
>Pyrus_hap1.log 2>&1
```

```
java -jar -Xmx10G
/path/to/juicer/juicer_tools.1.9.9_jcuda.0.8.jar pre
Pyrus_hap1.txt Pyrus_hap1.hic <(echo "assembly 540812361")
```

```
~/Alan_bin/yahs/juicer post -o Pyrus_hap1_v1.3
Pyrus_hap1_v1.2.11_27.assembly Pyrus_hap1_v1.2.liftover.agp
../../Pyrus_hap1_v1.2.rename.fasta
```

Dot plots between haplotypes and to *M. domestica* "Honeycrisp" were then regenerated to identify potential misassembled inversions as well as alternate assembled haplotypes. A large inversion was identified on haplotypes 1 scaffold 14. Omni-C contact maps suggest this was a misassembly.

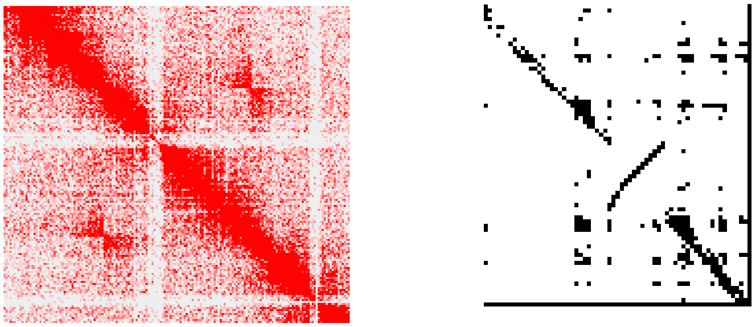

Figure SM9: (left) Omni-C contact map for haplotype 1 scaffold 14 showing a bowtie motif at a hypothesized assembly error. (right) dotplot between *Pyrus* and *Malus* showing a possible assembly error.

If this was a true inversion, the contact map would not show a bowtie motif.

Note: all of the manual edits going forward in our assembly are padded by 10,000 “N” characters for easy identification and reversion if necessary.

The hypothesized misassembly was corrected with a custom script to invert a given region. The breakpoints were identified in the dot plot generated from Gepard.

```
python ~/Alan_bin/python/invert_seq.py --input
Pyrus_hap1_scaff14.fasta --output Pyrus_hap1_scaff14_edit.fasta
--left_bound 24279111 --right_bound 25014233
```

An alternate assembled haplotype was identified in haplotype 1 scaffold 11. The Omni-C contact map supports this as a misassembly.

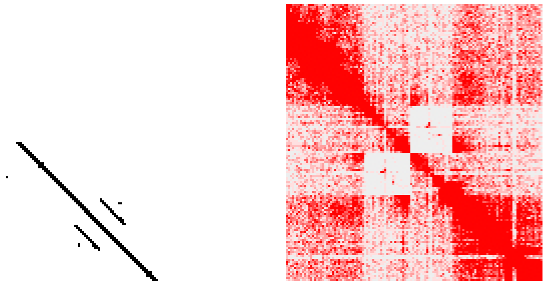

Figure SM10: (left) Self vs. self dotplot of haplotype 1 scaffold 11 shows a relatively large tandem duplication that is likely a 3rd assembled haplotype error. (right) Omni-C contact map of this region.

This region was removed using a custom script. Breakpoints were identified from the dot plots generated from Gepard.

```
python ~/Alan_bin/python/remove_alt_hap.py --input
Pyrus_hap1_scaff11.fasta --output Pyrus_hap1_scaff11_edit.fasta
--left_bound 2053728 --center_point 3628071 --right_bound 4163496
```

The final assembly file for haplotype 1 was `Pyrus_hap1_v1.2.11_28.assembly`. Note, the assembly files indicate which version of the genome Omni-C reads were mapped to. FASTA files generated from these assembly files are therefore one version more current than their name indicates. For example, `Pyrus_hap1_v1.2.11_28.assembly` was used to generate `Pyrus_hap1_v1.3.11_28.fasta`.

```
~/Alan_bin/yahs/juicer post -o Pyrus_hap1_v1.3
Pyrus_hap1_v1.2.11_28.assembly Pyrus_hap1_v1.2.liftover.agp
../../Pyrus_hap1_v1.2.rename.fasta
```

Scaffolds 11 and 14 in haplotype 1 were replaced with those with misassemblies corrected.

```
~/Alan_bin/perl/subset_fa.pl -s scaffold_11,scaffold_14 --invert -f
yahs/round_3/Pyrus_hap1_v1.3.FINAL.fa -o
Pyrus_hap1_v1.3._sub_seq.fasta
genes match line: 417
cat Pyrus_hap1_scaff11_edit.fasta >> Pyrus_hap1_v1.3._sub_seq.fasta
cat Pyrus_hap1_scaff14_edit.fasta >> Pyrus_hap1_v1.3._sub_seq.fasta
```

Scaffolds were then again renamed to match their homologs in *M. domestica* “Honeycrisp”.

```
~/Alan_bin/perl/rename_fa.pl -i hap1_HC_hap1_trans.txt -f
Pyrus_hap1_v1.3._sub_seq.fasta -o Pyrus_hap1_v1.3.rename.fasta
```

Dot plots were then generated of haplotype 2 to *M. domestica* “Honeycrisp”.

An alternative haplotype was identified in haplotype 2 scaffold 6.

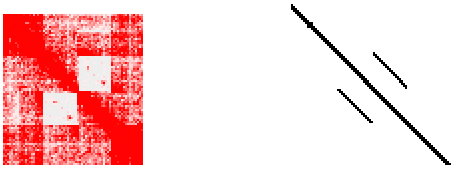

Figure SM11: (left) Omni-C contact map of haplotype 2 scaffold 6 in a hypothesized 3rd haplotype assembly error region. (right) self vs. self dotplot of haplotype 2 scaffold 6.

This region was removed using a custom script. Breakpoints were identified from the dot plots generated from Gepard.

```
 ~/Alan_bin/python/remove_alt_hap.py --input Pyrus_hap2_scaff6.fasta
--output Pyrus_hap2_scaff6_edit.fasta --left_bound 23563461
--center_point 24437043 --right_bound 25280395
```

There were two additional alternative haplotypes identified in haplotype 2 scaffold 5.

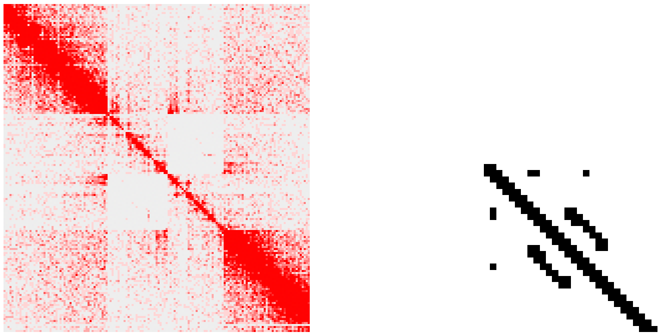

Figure SM12: (left) Omni-C contact map of haplotype 2 scaffold 5 in a hypothesized 3rd haplotype assembly error region. (right) self vs. self dotplot of haplotype 2 scaffold 5.

This region was removed using a custom script. Breakpoints were identified from the dot plots generated from Gepard.

```
~/Alan_bin/python/remove_alt_hap.py --input Pyrus_hap2_scaff5.fasta
--output Pyrus_hap2_scaff5_edit1.fasta --left_bound 28003564
--center_point 28296370 --right_bound 28569250
```

There was a second issue of the same nature on haplotype 2 scaffold 5:

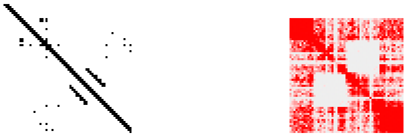

Figure SM11: (left) Omni-C contact map of haplotype 2 scaffold 5 in a hypothesized 3rd haplotype assembly error region. (right) self vs. self dotplot of haplotype 2 scaffold 5.

```
~/Alan_bin/python/remove_alt_hap.py --input
Pyrus_hap2_scaff5_edit1.fasta --output Pyrus_hap2_scaff5_edit2.fasta
--left_bound 11799692 --center_point 12202873 --right_bound 12634803
```

A FASTA formatted file was generated from Juicebox.

```
~/Alan_bin/yahs/juicer post -o Pyrus_hap1_v1.3
Pyrus_hap1_v1.2.11_28.assembly Pyrus_hap1_v1.2.liftover.agp
../../Pyrus_hap1_v1.2.rename.fasta
```

Scaffolds 6 and 5 in haplotype 2 were replaced with those where alternate assembled haplotypes were removed.

1.5 K-mer assessment of completeness

A Merqury analysis was performed for a *k*-mer based assessment of genome assembly quality.

```
#select best kmer size
sh $MERQURY/best_k.sh 700000000
genome: 700000000
tolerable collision rate: 0.001
19.6736
```

2. Annotation

2.1 Repeat identification and masking

2.2 RNA-seq filtering and alignment

RNA-seq reads were cleaned by filtering out adapter sequences and ribosomal kmers.

```
${bbdir}/bbduk.sh in=${r1} in2=${r2} out=tmp_R1.fq out2=tmp_R2.fq \
  ref=${bbdir}/ribokmers.fa.gz

  ${bbdir}/bbduk.sh in=tmp_R1.fq in2=tmp_R2.fq out=${out_base}_R1.fq
out2=${out_base}_R2.fq \
  ref=${bbdir}/TruSeq3-PE.fa minlength=25 ktrim=r
```

RNA-seq reads were mapped to each haplotype

```
#/cluster/home/ayocca/miniconda3/envs/genome_tools/bin/hisat2-align-s
version 2.2.1
#64-bit
#Built on fv-az212-765
#Wed Feb 23 05:52:46 UTC 2022
#Compiler: collect2: error: ld returned 1 exit status
#Options: -O3 -m64 -msse2 -funroll-loops -g3 -DPOPCNT_CAPABILITY
-std=c++11
#Sizeof {int, long, long long, void*, size_t, off_t}: {4, 8, 8, 8, 8,
8}
```

```
hisat2 -p 10 -x Malus_gs_hap1_v1.3.rename.hisat_idx \
-1 ${read_one} \
-2 ${read_two} \
-S ${output}

bam=$(echo $output | sed "s/sam/bam/")

#samtools 1.6
#Using htslib 1.6
#Copyright (C) 2017 Genome Research Ltd.

samtools sort -@ 10 ${output} > ${bam}
```

Bam files were merged.

```
samtools merge all_merged_hap1.bam *_clean_hap1.bam
```

A transcriptome was assembled to use as evidence for gene annotation.

```
#version 2.1.7
stringtie -p 9 -o all_merged_hap1.gtf all_merged_hap1.bam

#version 0.12.7
gffread Fruitlet_Stage_1.gtf -g Malus_gs_hap1_v1.3.rename.fasta -w
Fruitlet_Stage_1.fasta
```

Arabidopsis thaliana Araport11 proteins were also used as annotation evidence.

Each scaffold was annotated separately with three rounds of MAKER. The first round was based on evidence only. The second round included both the evidence plus *ab-initio* trained models. The third round of annotation included the original evidence and *ab-initio* models retrained on the second round of MAKER.

Control files are included in the scripts directory

ab-initio training was performed according to https://biohpc.cornell.edu/doc/annotation_2019_exercises1_v2.pdf.

```
#train snap
maker2zff -l 50 -x 0.5 chr1B_evi.all.gff
```

```
fathom -categorize 1000 genome.ann genome.dna
fathom -export 1000 -plus uni.ann uni.dna
forge export.ann export.dna
hmm-assembler.pl pyu . > ../chr1B_evi.hmm
```

For augustus optimization, the training set was subsetted to 2,000 genes to improve speed.

```
awk '{if ($2=="maker") print }' hap1_ab1.all.gff > hap1_ab1.maker.gff
gff2gbSmallDNA.pl hap1_ab1.maker.gff Pyrus_hap1_v1.3.rename.fasta
2000 hap1_ab1.gb
new_species.pl --species=hap1_ab1
etraining --species=hap1_ab1 hap1_ab1.gb
subset_gb.py --input hap1_ab1.gb --ngenes 2000 --output
hap1_ab1_sub.gb
randomSplit.pl hap1_ab1_sub.gb 200
optimize_augustus.pl --species=hap1_ab1 --kfold=24 --cpus=24
--rounds=3 --onlytrain=hap1_ab1_sub.gb.train hap1_ab1_sub.gb.test &>
hap1.log
```

The annotation files from three rounds of MAKER were named `Pyrus_hap1_v1.3.all.maker.transcripts.fasta` and `Pyrus_hap2_v1.3.all.maker.transcripts.fasta`. 68,452,305 bases were annotated in haplotype 1 across 44,839 gene models. 68,084,531 bases were annotated in haplotype 2 across 44,561 gene models.

BUSCO analysis was performed on the annotations and the results are as follows:

Haplotype	Complete	Single-copy	Duplicate	Fragmented	Missing	Total number
1	97.1%	63.1%	34.0%	1.3%	1.6%	1614
2	97.6%	64.3%	33.3%	0.9%	1.5%	1614

Table SM2: BUSCO values for *P. communis* “dAnjou” v1.3 annotations.

Gene models were renamed to match the following convention:
Pyrco.da.v2a1.ch01A.g000010.t1

```
maker_map_ids --prefix Pyrco.da.v2a1. --justify 5
Pyrus_hap1_v1.3.maker.gff > Pyrus_hap1.all.id.map
```

```
while read line; do old=$(echo $line | sed "s/ .*//g"); chrom=$(echo $old | sed "s/maker\-//g" | sed "s/\-.*//g"); new=$(echo $line | sed "s/.* //g" | sed "s/a1/a1.${chrom}/"); if [[ $new =~ .*[0-9] ]]; then new=$(echo $new | sed "s/$/0/g"); else new=$(echo $new | sed "s/\-RA/0.t1/" | sed "s/\-RB/0.t2/" | sed "s/\-RC/0.t3/" | sed "s/\-RD/0.t4/" | sed "s/\-RE/0.t5/"); fi; echo "${old}     x${new}" >> Pyrus_hap1.all.id.map.edit; done < Pyrus_hap1.all.id.map

map_fasta_ids Pyrus_hap1.all.id.map.edit Pyrus_hap1_v1.3.all.maker.proteins.fasta

map_gff_ids Pyrus_hap1.all.id.map.edit Pyrus_hap1_v1.3.maker.gff
```

3. Miscellaneous analyses

3.1 Chloroplast genome assembly

Publicly available DNA sequencing reads were collected using the sratoolkit v3.0.2 and listed in Table S8. Reads were subsetted to 1Gb of sequence by taking the first N reads from these files. The value of N changed with the length of the reads. The subsetted reads were assembled with Novoplasty v2.20 (Dierckxsens et al. 2017). An example configuration file `config_pyrifolia.txt` is provided. The chloroplast assembly NC_045336 was used as a reference. Not all chloroplasts were assembled as a single circular sequence so the first possible combination (eg `Option_1_pyrifolia.fasta`) was selected to annotate except for *Pyrus communis* cv. “Bartlett”. “Bartlett” option 2 was oriented consistently with option 1 of all other species. Chloroplasts were annotated using GEseq using default parameters. The following options were selected: circular, Plastid (land plants), Annotate plastid Inverted Repeat (IR), Annotate plastid trans-spliced *rps12*, Support annotation by Chloe, keep best annotation only, Protein search identity 25, rRNA tRNA DNA search identity 85, annotate CDS tRNA rRNA, ignore genes annotated as locus tag, MPI-MP Reference Set chloroplast land plats, Chloe v0.1.0 annotate CDS. Assemblies were visualized using OGDRAW (Greiner et al. 2019)

Each chloroplast assembly was shifted to begin with the *matK* gene as annotated by blatX. The chloroplast assemblies were then aligned using MAFFT to generate a maximum likelihood phylogeny with RAxML (Katoh and Standley 2013; Stamatakis 2014).

```
```

```
~/center_cp.py --start <matK_start_coord> --cp_fasta cp.fasta
--output cp_matK_start.fasta

cat *matK_start.fasta >> Pyrus_cp_matK_start.fasta

mafft"  --auto --inputorder "Pyrus_cp_matK_start.fasta" >
"Pyrus_cp_matK_start_aln.fasta

raxml-ng --msa Pyrus_cp_matK_start_aln.fasta --model GTR
```
